# Supplementary material for: IL-6 Responsiveness of CD4+ and CD8+ T Cells after Allogeneic Stem Cell Transplantation Differs between Patients and Is Associated with Previous Acute Graft versus Host Disease and Pretransplant Antithymocyte Globulin Therapy
Source: J Clin Med. 2022 Apr 30;11(9):2530. doi: 10.3390/jcm11092530 (PMC9104003; doi:10.3390/jcm11092530)
Supplement: Supplementary file 1 [file jcm-11-02530-s001.zip › jcm-1670506-supplementary.pdf]

SUPPLEMENTARY INFORMATION

IL-6 RESPONSIVENESS OF CD4$^+$ AND CD8$^+$ T CELLS AFTER ALLOGENEIC STEM CELL TRANSPLANTATION DIFFERS BETWEEN PATIENTS AND IS ASSOCIATED WITH PREVIOUS ACUTE GRAFT-VERSUS-HOST DISEASE AND PRETRANSPLANT ANTITHYMOCYTE GLOBULIN THERAPY

Tor Henrik Anderson Tvedt[1,2,3], Stephan Rose-John[4], Galina Tsykunova[2], Aymen Bushra Ahmed[2], Tobias Gedde-Dahl[1,6], Elisabeth Ersvær[6] and Øystein Bruserud[2,3]

[1] Department of Hematology, University of Oslo, 0424 Oslo, Norway.

[2] Section for Hematology, Department of Medicine, Haukeland University Hospital, Bergen, Norway.

[3] Section for Hematology, Institute of Clinical Science, University of Bergen, Bergen, Norway.

[4] Institute of Biochemistry, Kiel University, Olshausenstrasse 40, Kiel, Germany.

[5] Institute of Clinical Medicine, University of Oslo, Oslo, Norway

[6] Department of Biomedical Laboratory Scientist Education, Western Norway University of Applied Sciences, Bergen, Norway.

Supplementary Table S1. Intracellular signaling in $CD3^-$ mononuclear cells; a comparison of patients with and without previous acute GVHD. The table shows the effects of four forms of IL-6 stimulation on the phosphorylation of the intracellular mediators Akt(Thr308), mTOR(Ser2448), STAT3(Ser727) and STAT3(Tyr705) in $CD3^-$ circulating mononuclear cells. All p-values refer to a statistical comparison between cultures with and without IL-6 activation signals; the corresponding control cultures thus contained PMA alone or PMA+anti-CD3+anti-CD28. The table shows the combinations with increased mediator phosphorylation corresponding to $p<0.05$. All comparisons were done using the Wilcoxon Signed Rank Test.

**Phosphotarget**	**H-IL-6**	**IL-6**	**IL-6 sIL-6R**	**IL-6 sIL-6R sgp130FC**
		**All patients**		
**AKT(Thr308)**				
**mTOR(Ser2448)**				0.02
**STAT3(Ser727)**				
**STAT3(Tyr705)**	0.005			0.04
		**Patients with acute GVHD**		
**AKT(Thr308)**				
**mTOR(Ser2448)**				
**STAT3(Ser727)**				
**STAT3(Tyr705)**	0.02		0.04	
		**Patients without acute GVHD**		
**AKT(Thr308)**				
**mTOR(Ser2448)**				
**STAT3(Ser727)**				
**STAT3(Tyr705)**				

Supplementary Table S2. The effects of IL-6 stimulation on circulating CD3$^+$CD4$^+$ and CD3$^+$CD8$^+$ T cells derived from patients with and without ATG as a part of their pretransplant GVHD prophylaxis. The effects of four different forms of IL-6 stimulation on the phosphorylation of Akt(ThrR308), mTOR(Ser2448), STAT3(Ser727) and STAT3(Tyr705) were investigated. All p-values refer to a statistical comparison between cultures with and without IL-6 activation signals; the corresponding control cultures thus contained medium alone or anti-CD3+anti-CD28. The table shows the combinations with increased mediator phosphorylation corresponding to $p<0.05$.

	Without TCR ligation				With TCR ligation			
**Phosphotarget**	**H-IL-6**	**IL-6**	**IL-6 sIL-6R**	**IL-6 sIL-6R sgp130FC**	**H-IL-6**	**IL-6**	**IL6 sIL-6R**	**IL-6 sIL-6R sgp130**
**CD3$^+$CD4$^+$ T cells; patients treated with ATG**								
**AKT(Thr308)**								
**mTOR(Ser2448)**								0.04
**STAT3(Ser727)**	0.01	0.04						
**STAT3(Tyr705)**	0.002	0.007	0.007	0.004	<0.001	<0.001	<0.001	0.02
**CD3$^+$CD4$^+$ T cells; patients not treated with ATG**								
**AKT(Thr308)**								
**mTOR(Ser2448)**								0.02
**STAT3(Ser727)**					0.01		0.01	
**STAT3(Tyr705)**	<0.001	0.005	0.001	0.003	<0.001	<0.001	<0.001	<0.001
**CD3$^+$CD8$^+$ T cells; patients treated with ATG**								
**AKT(Thr308)**						0.02		
**mTOR(Ser2448)**								
**STAT3(Ser727)**	0.01	0.02						
**STAT3(Tyr705)**	0.002				0.01			
**CD3$^+$CD8$^+$ T cells, patients not treated with ATG**								
**AKT(Thr308)**								
**mTOR(Ser2448)**							<0.001	0.003
**STAT3(Ser727)**					0.02	0.03		
**STAT3(Tyr705)**					<0.01			

Supplementary Table S3. The effects of PMA stimulation on T cell subsets. The table shows the effects of PMA alone and PMA+anti-CD3+anti-CD28 on the phosphorylation of STAT3/Akt/mTOR in circulating CD3$^+$CD4$^+$ T cells, CD3$^+$CD8$^+$ T cells and CD3$^-$ mononuclear cells derived from allotransplant recipients treated with ATG or not treated with ATG. All p-values refer to a statistical comparison between cultures with and without PMA activation signals; the corresponding control cultures were prepared in medium alone. The table shows the stimulations with increased mediator phosphorylation corresponding to $p<0.05$. All comparisons were done using the Wilcoxon Signed Rank Test.

	**CD3$^+$CD4$^+$**		**CD3$^+$CD8$^+$**		**CD3$^-$**
**Phosphotarget**	**PMA alone**	**PMA anti-CD3 anti-CD28**	**PMA alone**	**PMA anti-CD3 anti-CD28**	**PMA alone**
**Patients treated with ATG**					
AKT(Thr308)		0.01	0.002	0.001	0.04
mTOR(Ser2448)	0.002	0.01	<0.0001	0.01	<0.001
STAT3(Ser727)	0.01		0.001		0.005
STAT3(Tyr705)	0.004				
**Patients not treated with ATG**					
AKT(Thr308)	<0.001	0.001	0.002	<0.001	0.03
mTOR(Ser2448)	<0.001	<0.001	<0.001	<0.001	<0.001
STAT3(Ser727)	<0.001	0.002	0.005	0.002	0.02
STAT3(Tyr705)		.			

Supplementary Table S4. Effects of IL-6 stimulation on intracellular signaling in $CD3^-$ mononuclear cells; a comparison of patients with and without previous ATG therapy. The effects of four forms of IL-6 stimulation on the phosphorylation of the intracellular mediators Akt(Thr308), mTOR(Ser2448), STAT3(Ser727) and STAT3(Tyr705) in $CD3^-$ circulating mononuclear cells were investigated. All p-values refer to a statistical comparison between cultures with and without IL-6 activation signals; the corresponding control cultures thus contained medium alone or anti-CD3+anti-CD28. The table shows the combinations with increased mediator phosphorylation corresponding to $p<0.05$. All comparisons were done using the Wilcoxon Signed Rank Test.

**Phosphotarget**	**H-IL-6**	**IL-6**	**IL-6<br>sIL-6R**	**IL-6<br>sIL-6R<br>sgp130**
**Patients treated with ATG**				
**AKT(Thr308)**				
**mTOR(Ser2448)**				0.03
**STAT3(Ser727)**				
**STAT3(Y705)**	0.007			
**Patients not treated with ATG**				
**AKT(Thr308)**				
**mTOR(Ser2448)**				
**STAT3(Ser727)**				
**STAT3(Y705)**				
